# Efficacy and safety of Shufeng Jiedu capsule for coronavirus disease 2019 (COVID-19): A protocol for systematic review and meta-analysis

**DOI:** 10.1097/MD.0000000000021615

**Published:** 2020-08-07

**Authors:** Runmin Li, Ying Li, Bingchen Li, Haiyang Sun, Xinyu Liu, Xin Ge, Yuanxiang Liu, Jiguo Yang

**Affiliations:** a College of Traditional Chinese Medicine, Shandong University of Traditional Chinese Medicine, Jinan; b Nanchang University, Jiangxi; c Department of Neurology, Affiliated Hospital of Shandong University of Traditional Chinese Medicine; d College of First Clinical Medicine, Shandong University of Traditional Chinese Medicine; e College of Acupuncture and Massage, Shandong University of Traditional Chinese Medicine, Jinan, People's Republic of China.

**Keywords:** COVID-19, Shufeng Jiedu capsule, protocol, systematic review, meta-analysis

## Abstract

**Background:**

From the end of 2019 to the present, coronavirus disease 2019 (COVID-19) has put considerable pressure on the worlds medical system and caused significant mortality and economic losses around the world. In China, the Shufeng Jiedu capsule has been widely used in the treatment of COVID-19, but there is still a lack of evidence-based medical evaluation.

**Methods:**

According to the retrieval strategies, randomized controlled trials (RCTs) on the Shufeng Jiedu capsule for COVID-19 were obtained from CNKI, WanFang, VIP, PubMed, Embase and Cochrane Library, regardless of publication date, or language. Studies were screened based on inclusion and exclusion criteria, and the Cochrane risk bias assessment tool was used to evaluate the quality of the studies. The meta-analysis was performed using RevMan 5.3 and STATA 14.2 software. Ultimately, the evidentiary grade for the results will be evaluated.

**Results:**

This study will evaluate the efficacy and safety of the Shufeng Jiedu capsule in the treatment of COVID-19 and provide a more reasonable choice of medication in clinical practice.

**Conclusion:**

Our findings will provide references for future clinical decision and guidance development.

**Registration:**

INPLASY registration number: INPLASY202070024.

## 1 Introduction

Coronavirus disease 2019 (COVID-19), named by WHO, first broke out in Wuhan, Hubei Province, China, in late 2019.^[1,2]^ Caused by the coronavirus SARS-CoV-2, COVID-19 is generally susceptible.^[3]^ According to statistics from Johns Hopkins, as of June 30, 2020, 10,302,867 cases of COVID-19 have been confirmed, and there have been over 505,517 deaths worldwide.

The clinical manifestations and prognosis of COVID-19 are quite different. Most of the patients with mild symptoms (approximately 80.9%) have a favorable prognosis, with the main manifestations of fever, fatigue, and dry cough, and a few are accompanied by nasal congestion, runny nose, sore throat and diarrhea. However, severe and critical patients (approximately 19.1%) have a poor prognosis. They have high fever after the onset of the disease, and dyspnea and hyponea occur 1 week later.^[4]^ COVID-19 has put considerable pressure on the worlds medical system and caused significant mortality and economic loss around the world. Therefore, the exploration of effective treatment has become a top priority.

Traditional Chinese medicine (TCM) holds that COVID-19 belongs to the category of “epidemic febrile diseases”, has rich theoretical accumulation and shows reliable efficacy in the treatment of COVID-19.^[5,6]^ According to existing studies, Shufeng Jiedu capsule can reduce the Clinical Pulmonary Infection Score (CPIS) and the disappearance time of fever, rale, cough, and wheezing, and improve the pulmonary function test score and the Activities of Daily Living (ADL) score.^[7,8]^ From the perspective of cytokines, it can downregulate high sensitivity C-reactive protein (hs-CRP), procalcitonin (PCT), serum interferon gamma (IFN-γ), and interleukin-4 (IL-4).^[9]^ As an empirical drug, Shufeng Jiedu capsule is widely used to treat mild COVID-19, but its efficacy lacks evidence-based medical evaluation. Thus, we propose a protocol of systematic review to evaluate the efficacy and safety of Shufeng Jiedu capsule for COVID-19.

## 2 Methods

Our protocol has been registered on the International Platform of Registered Systematic Review and Meta-Analysis Protocols (INPLASY). The registration number was INPLASY202070024 (DOI: 10.37766/inplasy2020.7.0024). We strictly abide by Preferred Reporting Items for Systematic Review and Meta-Analysis Protocols (PRISMA-P) guidelines.^[10]^

### 2.1 Data sources and retrieval strategy

Studies were obtained from the China National Knowledge Infrastructure (CNKI), Wan Fang Data, Chinese Scientific Journals Database (VIP), PubMed, Embase and Cochrane Library, regardless of publication date or language.

The databases were searched by combining the subject words with random words. The retrieval strategy is shown in Table 1 using PubMed retrieval as an example. The search terms were adapted appropriately to conform to different syntax rules of different databases.

**Table 1:**
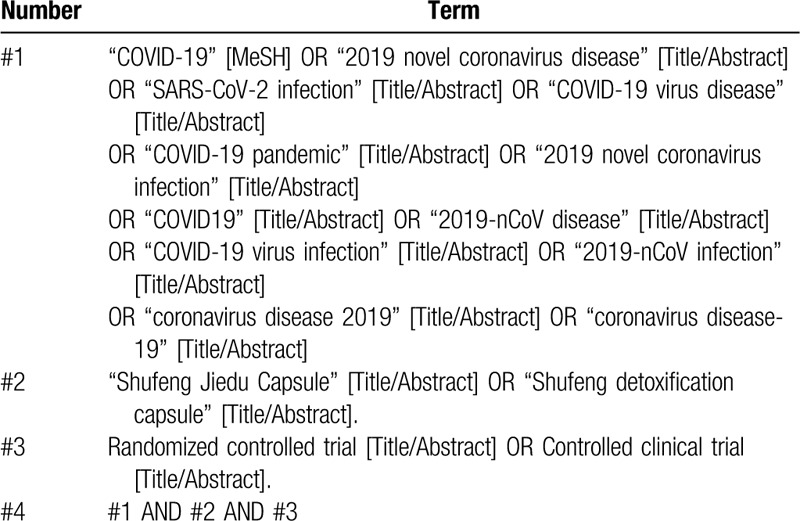
Retrieval strategy of PubMed.

### 2.2 Eligibility criteria

The PICOS principles were given full consideration to establish the inclusion and exclusion criteria of this systematic review.

#### 2.2.1 Type of participants

Regardless of age or gender, patients with COVID-19 met the diagnostic criteria of the National Health and Health Commission of the People's Republic of China on the diagnosis and treatment of new coronavirus infection pneumonia (trial version 7).^[3]^ Specifically, confirmation of a case had to meet one of the following criteria: positivity for novel coronavirus nucleic acid by real-time fluorescence RT-PCR; viral gene sequencing results that are highly homologous to the known novel coronavirus; and positivity for serum novel coronavirus-specific IgM and IgG antibodies.

#### 2.2.2 Type of interventions and comparators

The treatment group was given Shufeng Jiedu capsule on the basis of conventional treatment. The control group was only given routine treatment. Routine treatment mainly includes antiviral therapy, such as oral lopinavir, ritonavir, remdesivir, chloroquine, hydroxychloroquine, and arbidol.^[4]^

#### 2.2.3 Type of outcomes

The primary outcomes included the disappearance time of fever, rale, cough, wheezing, pulmonary function test score, and mortality rate.

Additional outcomes included CPIS, ADL, hs-CRP, PCT, IFN-γ, IL-4, white blood cell count, and incidence of adverse events.

#### 2.2.4 Type of studies

The included studies were RCTs in this systematic review regardless of publication status and language. Animal trials, clinical experience, case reports, and studies with wrong designs or incomplete data were excluded.

### 2.3 Study selection and data extraction

**Figure 1:**
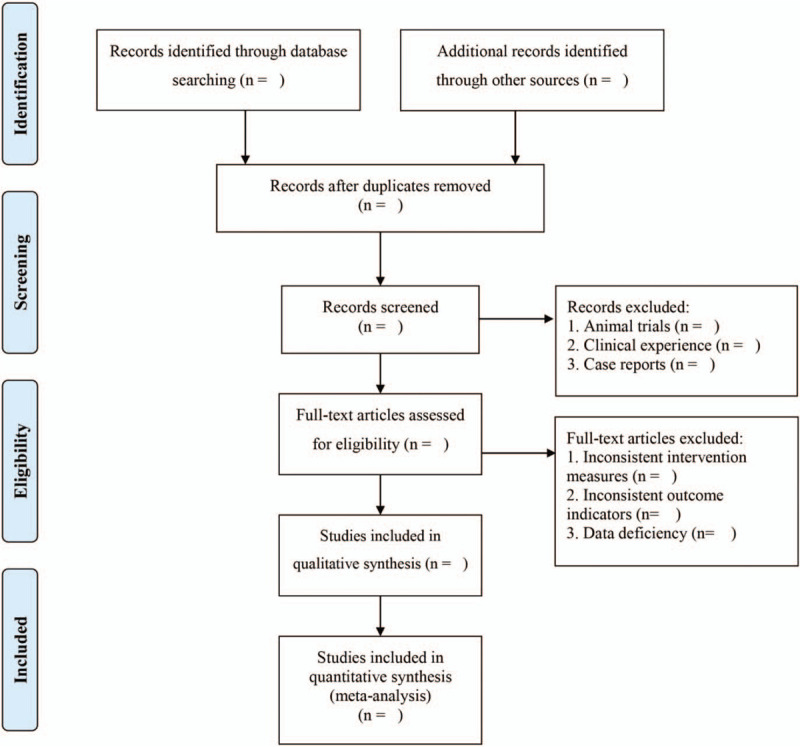
PRISMA flow chart.

EndNote X9 was used to manage the retrieved studies. As shown in Figure 1, the study selection was divided into 2 steps, which were completed by 2 researchers (Bingchen Li and Haiyang Sun). Preliminary screening involved eliminating repeated and unqualified studies by reading the title and abstract. Rescreening involved reading through the full text and selecting the studies according to the inclusion and exclusion criteria.

According to the Cochrane Handbook for Systematic Reviews of Intervention, the 2 researchers (Xinyu Liu and Xin Ge) extracted the author, publication time, participant number, age, sex, intervention measures, course of disease/treatment, and outcome indicators, filled in the data extraction table, and compared them with each other.

### 2.4 Risk of bias assessment

Two researchers (Bingchen Li and Xin Ge) assessed the quality of the included RCTs independently by utilizing the Cochrane risk of bias assessment tool. As specified by Cochrane Handbook V.5.1.0, the following sources of bias were considered: random sequence generation, allocation concealment, participant blinding, outcome assessor blinding, incomplete outcome data, selective reporting, and other sources of bias. Each domain was rated as having a high, low or unclear risk of bias as appropriate.^[11]^ The 2 reviewers resolved any disagreements through discussion, and a third reviewer (Runmin Li) was involved if a consensus could not be reached.

### 2.5 Statistical analysis

The meta-analysis was performed with Review Manager 5.3 and STATA 14.2 software. The outcomes were mainly represented by the mean difference (MD) or odds ratio (OR) with 95% confidence intervals, and a *P* value <.05 was considered significant. The Cochrane Q-test and *I*^2^ statistics were used to assess heterogeneity. When *P* < .1 or *I*^2^ > 50% indicated statistical heterogeneity, a random effects model was used to calculate the outcomes; otherwise, the fixed effect model was considered.

### 2.6 Subgroup analysis and publication biases

If there was high heterogeneity in the studies, we performed subgroup analyses to explore the differences in age, sex, interventions, and course of disease/treatment.

We used funnel plots to identify whether there was small study bias if 10 or more studies were included. The asymmetry of funnel plots suggests the possibility of small study effects, and the results of analysis were explained cautiously.

### 2.7 Sensitivity analysis

To ensure robustness of the combined results, sensitivity analyses were performed to assess the impact of studies with a high risk of bias. We compared the results to determine whether lower-quality studies should be excluded.

### 2.8 Quality of evidence

The Grading of Recommendations, Assessment, Development and Evaluation (GRADE) approach were used in evaluating evidence quality. Considerations of evidence quality assessment include study limitation, consistency of effect, imprecision, indirectness, and publication bias. The evidence quality was classified into 4 levels (high, medium, low, and very low).^[12]^

### 2.9 Ethics and Dissemination plans

Given that there are no patients recruited and no data gathered from patients, ethical approval is not necessary for our research. We will publish the results of this meta-analysis in the form of journal papers or conference papers.

## 3 Discussion

The TCM theory of “preventive treatment of disease” emphasizes “prevention before disease onset, rescuing disease in its germination and preventing disease from exacerbating”, that is, providing timely treatment in the early stages of the disease. Mild COVID-19 is the most common form of infection and can either infect others or become severe and should be taken seriously. As an adjunct therapy of TCM, the Shufeng Jiedu capsule has been used in the clinical treatment of COVID-19 and been reported to be effective, but the lack of evidence-based medical evaluation limits its wide application.^[13]^ Our study on the efficacy and safety of Shufeng Jiedu capsule for COVID-19 will provide a more reliable basis for future clinical decision and guidance development.

## Author contributions

**Conceptualization:** Runmin Li, Ying Li, Yuanxiang Liu and Jiguo Yang.

**Data curation:** Bingchen Li, Haiyang Sun, Xinyu Liu, Xin Ge.

**Formal analysis:** Runmin Li and Ying Li.

**Methodology:** Yuanxiang Liu and Jiguo Yang.

**Software:** Bingchen Li and Haiyang Sun.

**Supervision:** Yuanxiang Liu and Jiguo Yang.

**Writing – original draft:** Runmin Li and Ying Li.

**Writing – review & editing:** Yuanxiang Liu and Jiguo Yang.
